# Supplementary material for: Landscape Analysis of COL6A1, COL6A2, and COL6A3 Pathogenic Variants in a Large Italian Cohort Presenting with Collagen VI-Related Myopathies: A Nationwide Report
Source: Biomolecules. 2025 Oct 8;15(10):1426. doi: 10.3390/biom15101426 (PMC12564197; doi:10.3390/biom15101426)
Supplement: Supplementary file 1 [file biomolecules-15-01426-s001.zip › biomolecules-3788021-supplementary.pdf]

Table S1: Summary of VUS Characteristics in *COL6A1*, *COL6A2*, and *COL6A3* genes.

ID	M / F	MA/ BA	Genotype	Exon / Intron	cDNA change	Protein change	Domain	Mutation type	*In silico* prediction	ACMG criteria
COL6A1										
**UCMD**										
**P13**	M	MA	het	intron 10	c.904-10G>A	p.Gly302_Lys310del	THD	splicing	VUS	PM2, PP3
**P19**	M	BA	homo	exon 24	c.1576G>A	p.Gly526Arg	THD	missense	VUS	PM2, PP3, PP5
**BM**										
**P42**	M	MA	het	intron 11	c.931-3C>G	p.?	THD	splicing	VUS	PM2, PP3
COL6A2										
**ID**	**M / F**	**MA/ BA**	**Genotype**	**Exon / Intron**	**cDNA change**	**Protein change**	**Domain**	**Mutation type**	***In silico* prediction**	**ACMG criteria**
**UCMD**										
**P53**	M	MA	het	intron 9	c.954+23_955-43del	p.Gly310_Lys318del	THD	deletion	VUS	PM2
**P58**	M	BA	comp. het	exon 26	c.2098G>A	p.Gly700Ser	C (vWFA2)	missense	P	
				exon 26	c.2381C>A	p.Ala794Asp	C (vWFA2)	missense	VUS	PM2, PP3
**P61**	F	MA	het	exon 28	c.2560C>T	p.Arg854Cys	C (vWFA3)	missense	VUS	PM2
**INT**										
**P64**	M	MA	het	intron 5	c.801+3A>C	p.Cys246_Lys267del	N-THD	splicing	VUS	PM2, PP3
**INT/BM**										
**P74**	M	MA	het	exon 26	c.2060T>C	p.Phe687Ser	C (vWFA2)	missense	VUS	PM2, PP3
**BM**										
**P85**	F	BA	comp. het	exon 25	c.1832G>A	p.Cys611Tyr	C	missense	VUS	PM2, PP3
				exon 26	c.2329T>C	p.Cys777Arg	C (vWFA2)	missense	LP	
**P88**	M	MA	het	intron 25	c.1970-3C>A	p.Thr656_Ala698del	C (vWFA2)	splicing	VUS	PP3, PP5
**P91**	F	BA	comp. het	intron 25	c.1970-9G>A	p.Thr656fsTer18	C (vWFA2)	splicing	P	
				exon 28	c.2489G>A	p.Arg830Gln	C	missense	LP	
				exon 28	c.2527C>T	p.Arg843Trp	C (vWFA3)	missense	VUS	PM2, PP3
**P92**	M	BA	homo	exon 26	c.2060T>C	p.Phe687Ser	C (vWFA2)	missense	VUS	PM2, PP3
**P97**	M	BA	homo	exon 26	c.2240T>A	p.Leu747Gln	C (vWFA2)	missense	VUS	PM2, PP3
**P99**	M	MA	het	exon 28	c.2528G>A	p.Arg843Gln	C (vWFA3)	missense	VUS	PM2, PP3

**P100**	F	BA	comp. het	exon 28	c.2947_2952del	p.Asp983_Val984del	C (vWFA3)	deletion	VUS	PM2, PM4
				intron 1	chr21 g.(46352739_46352798)_(46354892_46354936)del Genome Build NCBI35/hg17 *	p.?		deletion	VUS	PM2
					COL6A3					
**ID**	**M / F**	**MA/ BA**	**Genotype**	**Exon / Intron**	**cDNA change**	**Protein change**	**Domain**	**Mutation type**	***In silico* prediction**	**ACMG criteria**
					**UCMD**					
**P108**	M	MA	het	intron 16	c.6210+5G>A	p.?	THD	splicing	VUS	PM2, PP3, PP5
**P113**	M	BA	comp. het	exon 32	c.7066G>A	p.Gly2356Arg	THD	missense	LP	
				intron 36	c.7669-3C>G	p.?		splicing	VUS	PM2, PP3
					**INT/BM**					
**P115**	F	MA	het	exon 7	c.2536G>A	p.Ala846Thr	N (vWFA5)	missense	VUS	PM2
**P116**	M	MA	het	exon 9	c.4121A>T	p.Asp1374Val	N (vWFA7)	missense	VUS	PM2, PP3
**P121**	F	MA	het	exon 36	c.7254C>A	p.Phe2418Leu	C (vWFA11)	missense	VUS	PM2, PP3
					**BM**					
**P123**	M	MA	het	exon 10	c.4859C>T	p.Pro1620Leu	N	missense	VUS	PM2
**P137**	F	MA	het	exon 28	c.6820G>A	p.Glu2274Lys	THD	missense	VUS	PM2, PP3
**P138**	M	MA	het	exon 36	c.7468G>A	p.Ala2490Thr	C (vWFA11)	missense	VUS	PM2

*GRCh37/hg19: g.47528311_47528370)_(47530470_47530508); M: Male; F: Female; MA: Monoallelic; BA: Biallelic; Het: heterozygous; Homo: homozygous; Comp het: compound heterozygous; UCMD: Ullrich congenital muscular dystrophy; INT: intermediate collagen VI-related myopathy; INT/BM: intermediate collagen VI-related myopathy/Bethlem myopathy; BM: Bethlem myopathy.
